# Supplementary material for: Melting domain size and recrystallization dynamics of ice revealed by time-resolved x-ray scattering
Source: Nat Commun. 2023 Jun 14;14:3313. doi: 10.1038/s41467-023-38551-0 (PMC10267142; doi:10.1038/s41467-023-38551-0)
Supplement: Supplementary file 1 — Supplementary Information [file 41467_2023_38551_MOESM1_ESM.pdf]

Supplementary Information for "Melting Domain Size and Recrystallization Dynamics of Ice Revealed by the Time-Resolved X-ray Scattering"

Cheolhee Yang[1], Marjorie Ladd-Parada[2], Kyeongmin Nam[1], Sangmin Jeong[1], Seonju You[1], Alexander Späh[2], Harshad Pathak[2], Tobias Eklund[2], Thomas J. Lane[3], Jae Hyuk Lee[4], Intae Eom[4], Minseok Kim[4], Katrin Amann-Winkel[2], Fivos Perakis[2], Anders Nilsson[2], and Kyung Hwan Kim[1,*]

[1]Department of Chemistry, Pohang University of Science and Technology (POSTECH), Pohang, Gyeongbuk 37673, Republic of Korea.

[2]Department of Physics, AlbaNova University Center, Stockholm University, SE-106 91 Stockholm, Sweden.

[3]SLAC National Accelerator Laboratory, 2575 Sand Hill Road, 'Menlo Park, CA 94025, USA.

[4]Pohang Accelerator Laboratory, Pohang, Gyeongbuk 37673, Republic of Korea.

[*]Corresponding author. Email: kimkyunghwan@postech.ac.kr

Supplementary Discussion

Results from the base temperature of 117 K

Experimental data at a base temperature of 117 K was collected and analyzed using the same protocol described in the "Methods" section of the main text. The results are summarized in Supplementary Figures 7 and 8. Although the number of delay point and statistics of the data are limited, the results appear to be consistent with those of the measurement at a base temperature of 170 K. The difference intensities, both in the SAXS and WAXS regions, show similar shape and time-dependent trends to those of the measurement at a base temperature of 170 K, indicating a similar melting and recrystallization process occurring in the sample. The relatively smaller molten fraction and SAXS intensity at this temperature compared with the case of 170 K are consistent with the effect of the lower base temperature. Additionally, it is shown that the decay of the SAXS intensity is slower than that of the melt thickness, indicating coalescence between the adjacent domains.

Supplementary Figures

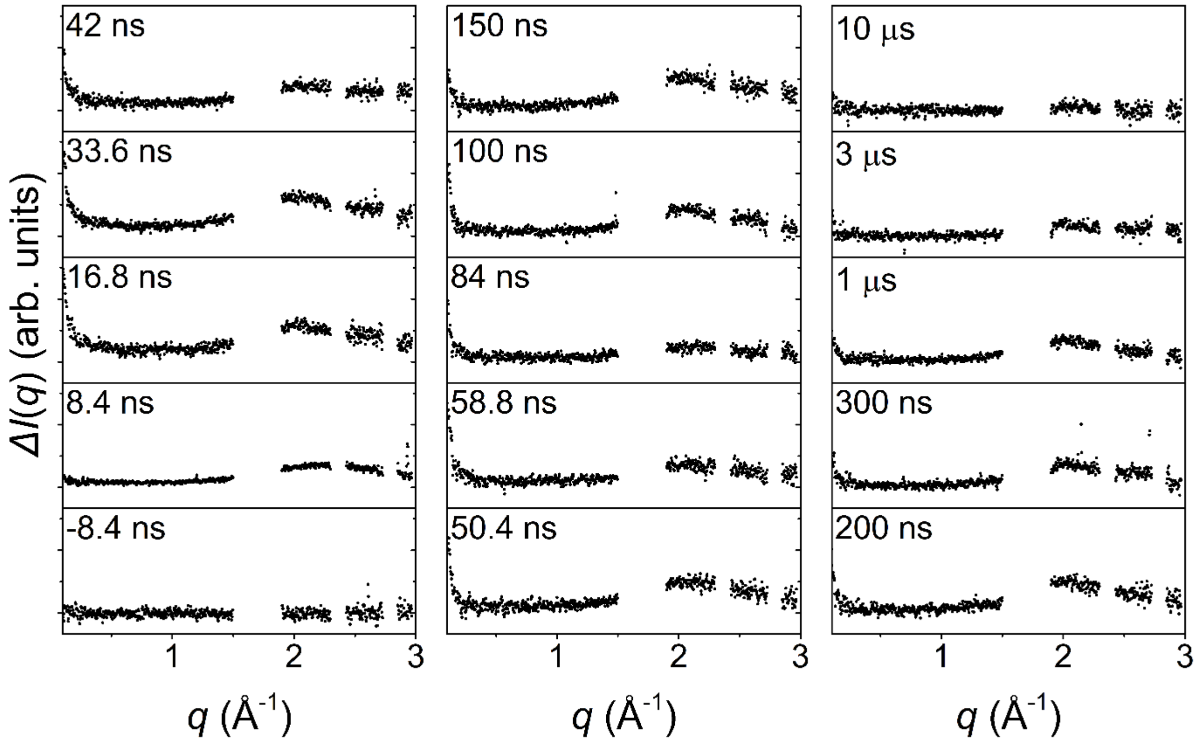

Supplementary Figure 1. The difference scattering intensities, Δ*I*(*q*), measured at various time delays from -8.4 ns to 10 μs. The regions nearby the Bragg diffraction peaks are not shown for clarity.

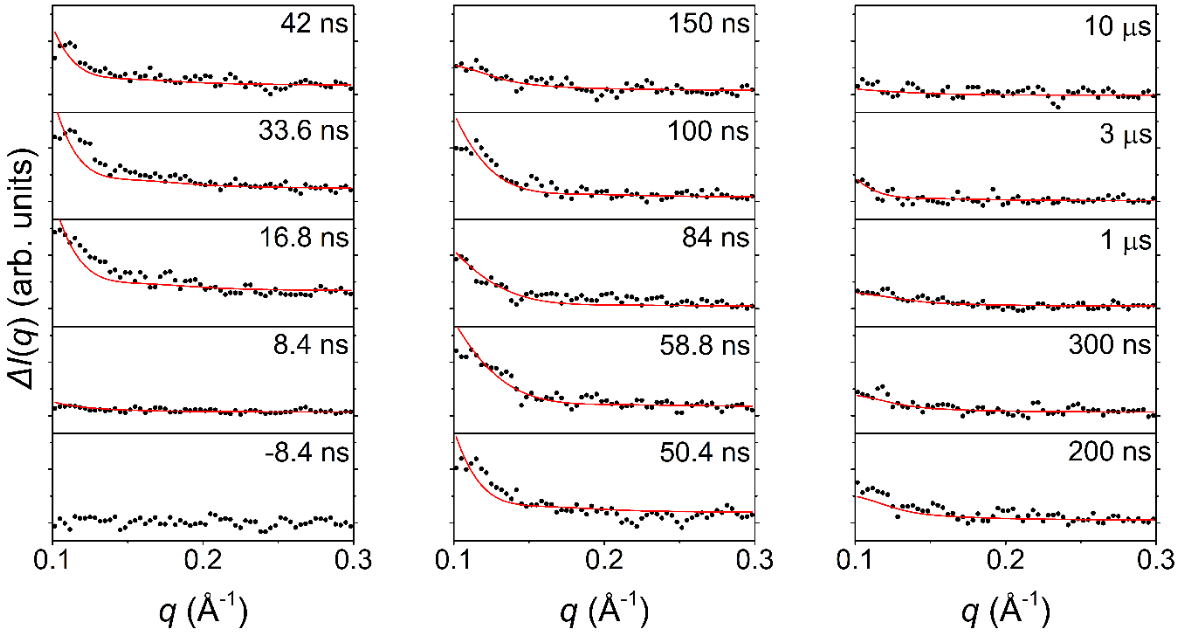

Supplementary Figure 2. The difference scattering intensities, $\Delta I(q)$, in the SAXS region at time delays from -8.4 ns to 10 µs (black) are shown together with the fitting results (red line).

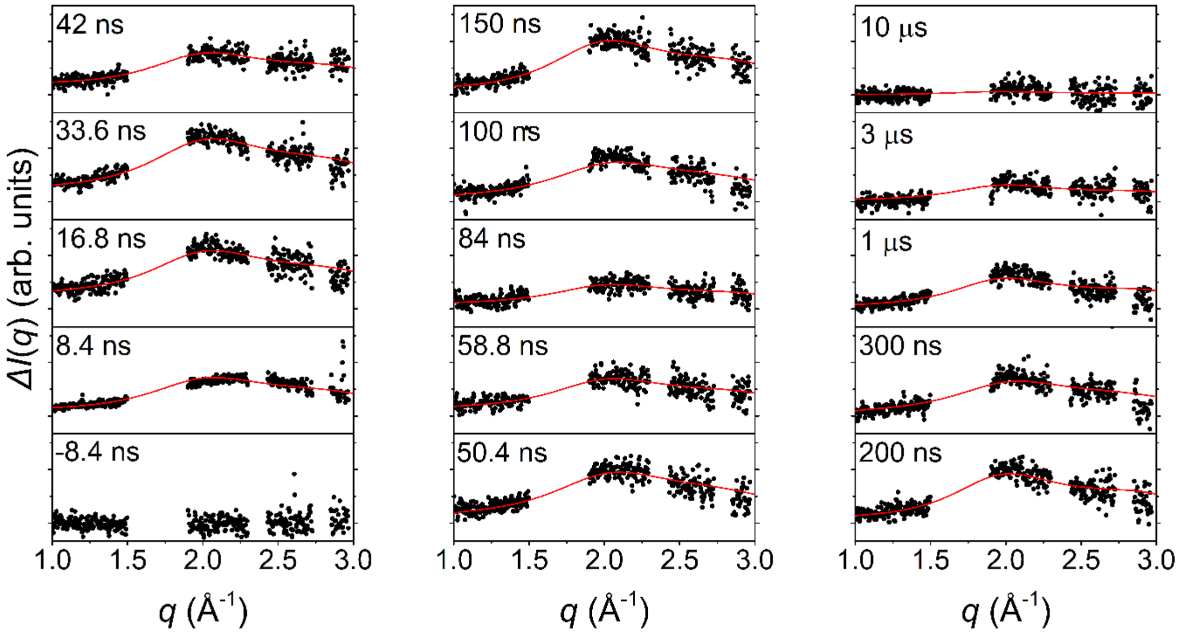

Supplementary Figure 3. The difference scattering intensities, $\Delta I(q)$, in the WAXS region at time delays from -8.4 ns to 10 μs (black circle) are shown together with their best match with the the scaled scattering pattern of liquid water from the reference among various temperatures from 254 K to 366 K[1]. The regions nearby the Bragg diffraction peaks are not shown for clarity.

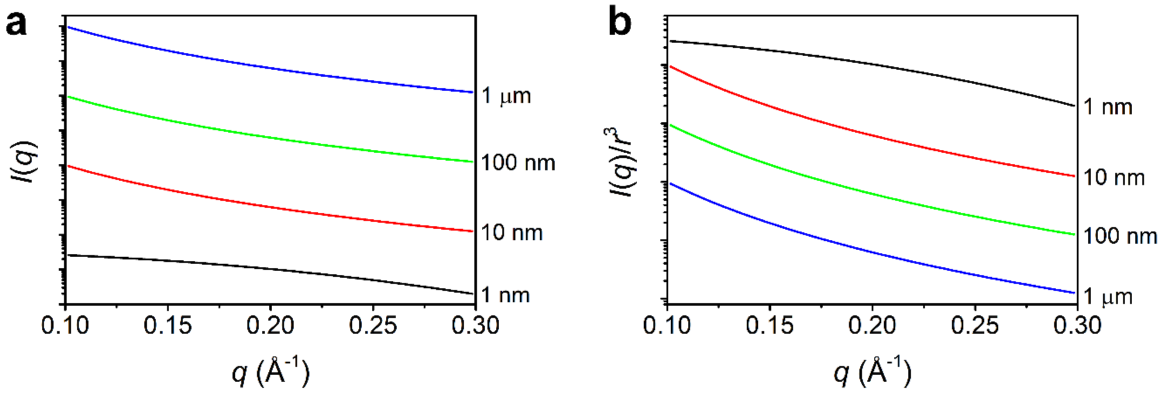

Supplementary Figure 4. Simulated SAXS intensities of liquid water domains of different radius (a) before and (b) after the normalization by r^3.

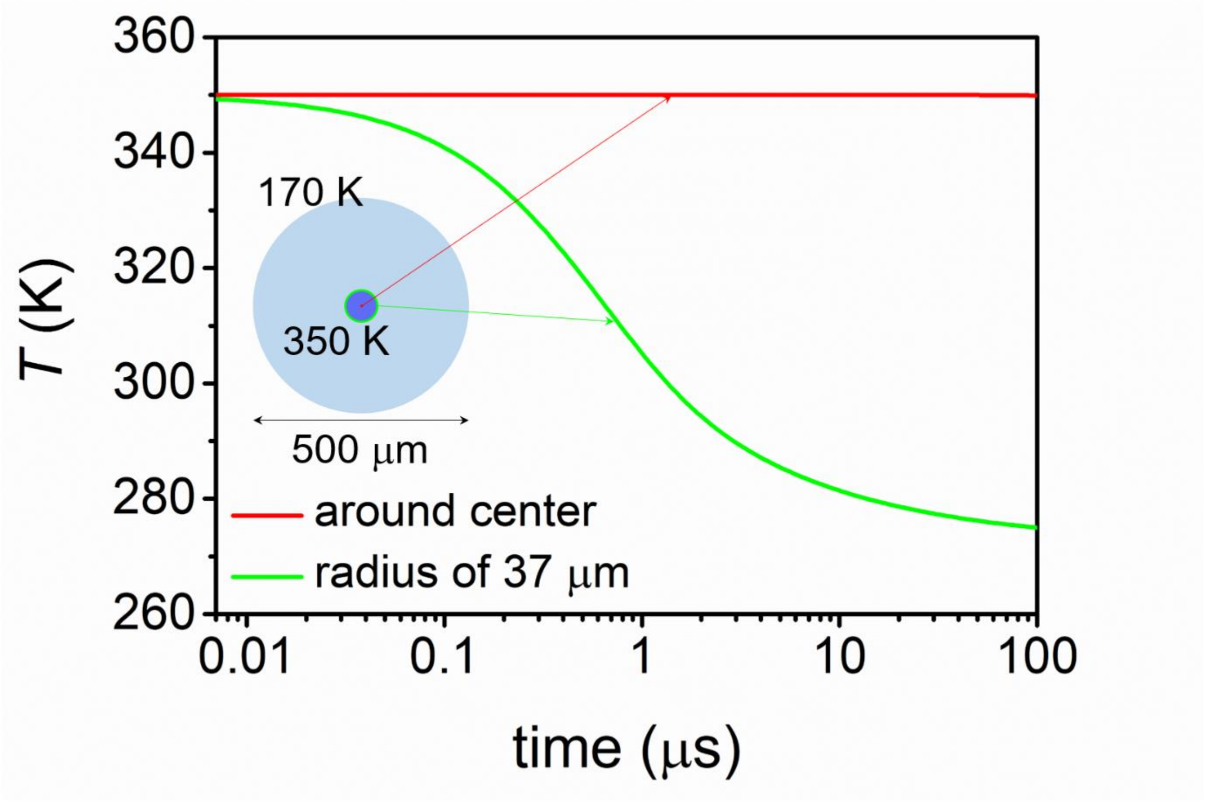

Supplementary Figure 5. Estimated timescales for heat dissipation in the sample. The time-depenent changes of the temperature near the center (red) and edge (green) of the irradiated area are shown.

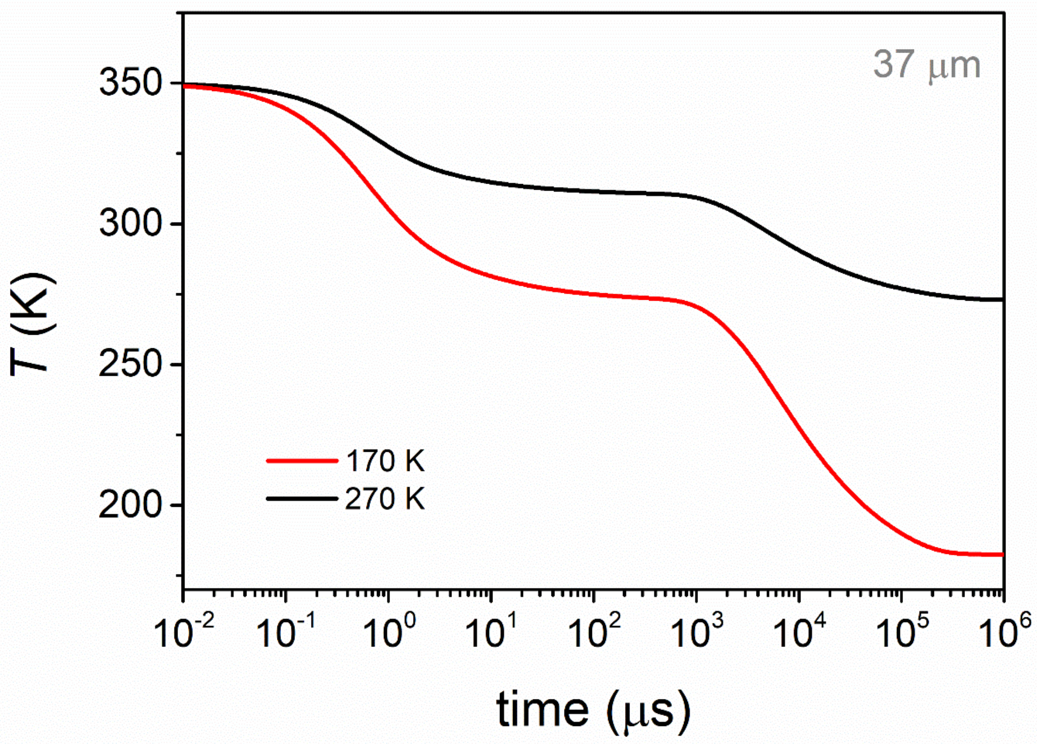

Supplementary Figure 6. Estimated timescales for heat dissipation in the sample with the base temperature of 270 K (black) and 170 K (red) near the edge of the irradiated area.

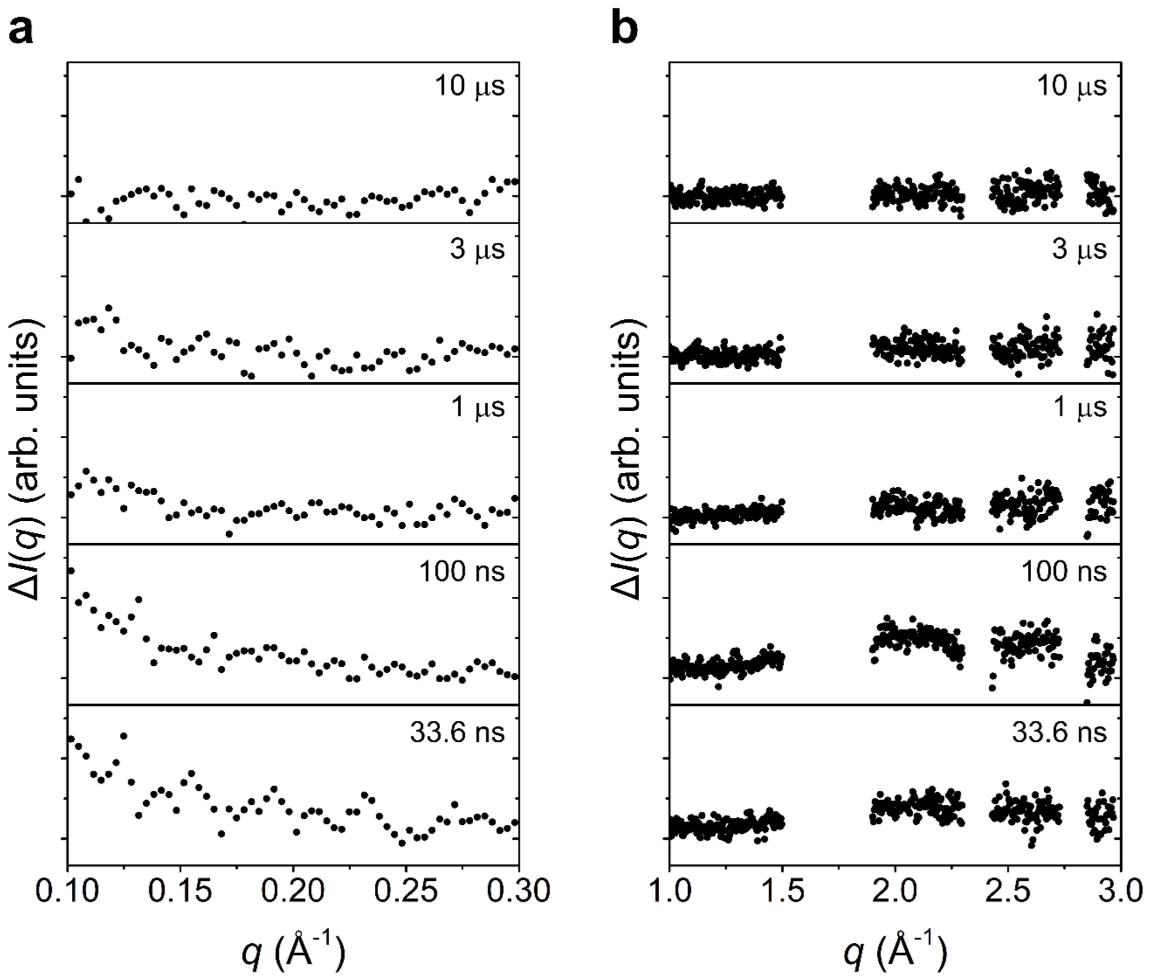

Supplementary Figure 7. The difference scattering intensities (black circles) in the (a) SAXS and (b) WAXS regions measured with the base temperature of 117 K.

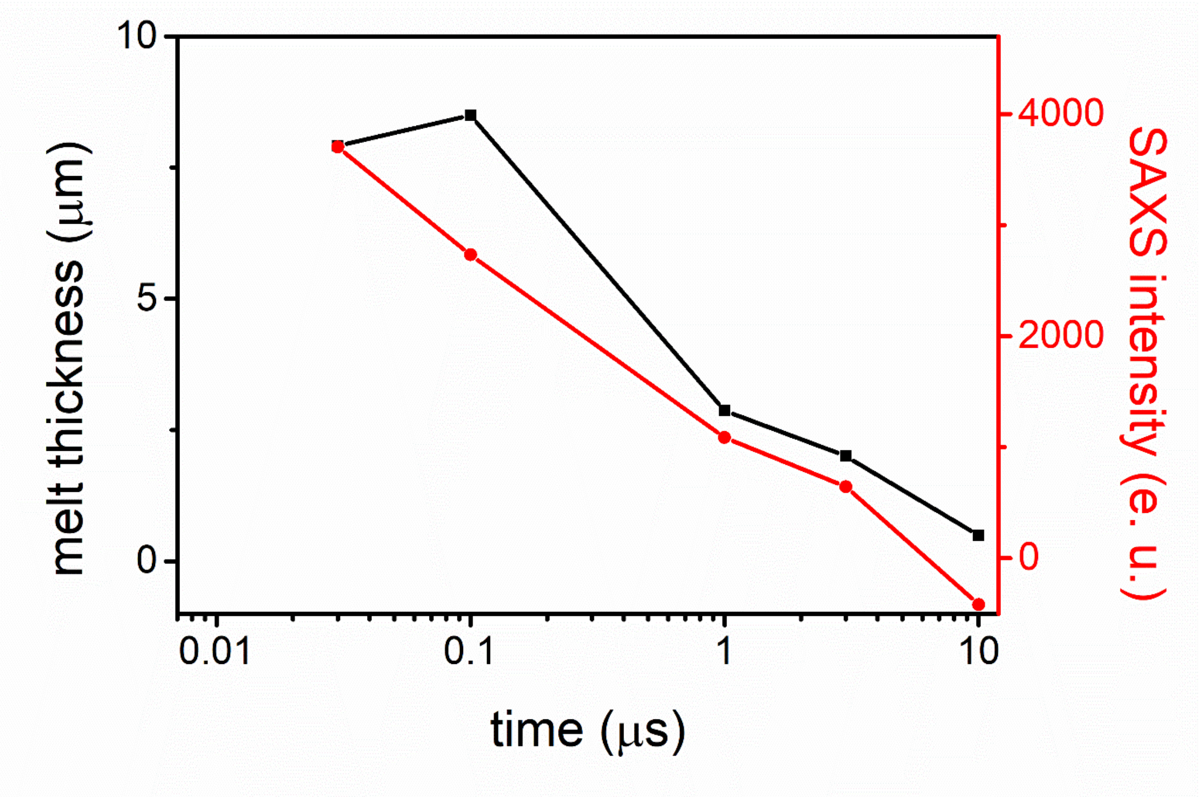

Supplementary Figure 8. The time-dependent change of the thickness of the liquid domain (black square) and the integrated intensity of the SAXS region of the difference scattering pattern (red circle) measured with the base temperature of 117 K. The unit for the SAXS intensity, e. u. means electron units.

Supplementary References

1. Skinner, L. B., Benmore, C. J., Neuefeind, J. C. & Parise, J. B. The structure of water around the compressibility minimum. *J. Chem. Phys.* **141**, 214507.
