# Supplementary material for: Melting domain size and recrystallization dynamics of ice revealed by time-resolved x-ray scattering
Source: Nat Commun. 2023 Jun 14;14:3313. doi: 10.1038/s41467-023-38551-0 (PMC10267142; doi:10.1038/s41467-023-38551-0)
Supplement: Supplementary file 3 — Peer Review File [file 41467_2023_38551_MOESM3_ESM.pdf]

nature portfolio

Peer Review File

Melting Domain Size and Recrystallization Dynamics of Ice Revealed by Time-Resolved X-ray Scattering

Open Access This file is licensed under a Creative Commons Attribution 4.0 International License, which permits use, sharing, adaptation, distribution and reproduction in any medium or format, as long as you give appropriate credit to the original author(s) and the source, provide a link to the Creative Commons license, and indicate if changes were made. In the cases where the authors are anonymous, such as is the case for the reports of anonymous peer reviewers, author attribution should be to 'Anonymous Referee' followed by a clear attribution to the source work. The images or other third party material in this file are included in the article's Creative Commons license, unless indicated otherwise in a credit line to the material. If material is not included in the article's Creative Commons license and your intended use is not permitted by statutory regulation or exceeds the permitted use, you will need to obtain permission directly from the copyright holder. To view a copy of this license, visit http://creativecommons.org/licenses/by/4.0/.

REVIEWER COMMENTS

Reviewer #1 (Remarks to the Author):

Yang and coworkers present an impressive experimental study of the melting and recrystallisation of water. From ice to liquid to ice again.

The process is resolved at the nanosecond scale and extends up to 100 microsends. Four order of magnitude in time, followed with two sophisticated techniques, SAXS and WAXS and an ingenious experimental set-up.

This study deserves to be published, but it requires in my opinion some work to better explain the relevance of their findings. In particular I believe the authors should discuss how the results are expected to depend on the intensity of the pulse (or else the initial average temperature) and if the sequence of the transformation (melting, coalescence, recrystallisation) is universal or if it is expected to be modified by the chosen experimental protocol. Similarly how the the size of the droplets, the size of the coalesced droplets, the time-scale of the different processes is expected to depend on the

experimental set-up ? I understand that it is not easy to repeat this type of complex experiments, requiring several shifts at the synchrotron facilities. But at least a comment on the expected generality of the results is highly welcome.

For example, would it be possible to tune, in a future study, the intensity of the IR beam to investigate crystal superheating or to bring the entire crystal close to its limit of stability ?

Further points include.

1) The error on the estimate od the temperature is probably of the order of the spreading of the points in Fig. 2b. This should be made clear by adding an error-bar. Even better, if would be nice to see a set of experimental data (as in Fig. 1d) but

with the best "fit" liquid s(q) and with the liquid s(q) at, let's say, 10 kelvin higher and lower.

2) The fit of the SAXS data is not really perfect at very low q (Fig. 1c). The authors should comment on this imperfection. Does it arise from experimental error in the subtraction procedure ? Or does it signal a real problem with the functional form ?

3) Eq.1 is nothing more than the form factor of a sphere. Why not to say it directly in the text ? This would make it easier for the reader who is not an expert in scattering.

In summary, this is an excellent experimental study which does deserve publication in Nature Communication. The authors show that nowadays, with cutting edge experiments, it becomes possible to access the very short timescale of melting and crystallisation. Still, the authors should make a significant effort to better explain to the reader the generality of their findings and how general are the "numbers" they have been able to evaluate for the first time.

Reviewer #2 (Remarks to the Author):

This paper uses a combination of time-resolved WAXS and SAXS to reveal structural changes in the time range from 0.01 to 10 microseconds in the melting and re-crystallization processes of ice. The authors monitor the growth or disappearance of the liquid domain in partially melted ice. The obtained data by using the time-resolved X-ray scattering techniques are significantly valuable in understanding the unique nature of water. I think that the paper should be published. However, some modifications seem to be needed. My comments and questions are as follows:

1. WAXS data is expected to contain hydrogen bonding information. The authors extracted only the thickness of the liquid phase from the WAXS information. Is it possible to extract hydrogen bonding information?

2. In Figure 2b, the dashed line could be misinterpreted as a theoretical value.

Is it possible to show it with S5?

3. Error bars are necessary in Figs. 2(b) and 3(b).

4. Fig.3 (a) shows the theoretical curves. Why are some spike-like noises seen?

Response to referees

Reviewer #1:

Yang and coworkers present an impressive experimental study of the melting and recrystallisation of water. From ice to liquid to ice again.

The process is resolved at the nanosecond scale and extends up to 100 microsends. Four order of magnitude in time, followed with two sophisticated techniques, SAXS and WAXS and an ingenious experimental set-up.

This study deserves to be published, but it requires in my opinion some work to better explain the relevance of their findings. In particular I believe the authors should discuss how the results are expected to depend on the intensity of the pulse (or else the initial average temperature) and if the sequence of the transformation (melting, coalescence, recrystallisation) is universal or if it is expected to be modified by the chosen experimental protocol. Similarly how the the size of the droplets, the size of the coalesced droplets, the time-scale of the different processes is expected to depend on the experimental set-up ? I understand that it is not easy to repeat this type of complex experiments, requiring several shifts at the synchrotron facilities. But at least a comment on the expected generality of the results is highly welcome.

For example, would it be possible to tune, in a future study, the intensity of the IR beam to investigate crystal superheating or to bring the entire crystal close to its limit of stability ?

→ We appreciate the reviewer's valuable comments and questions. We agree with the reviewer that addressing the generality of new results is important. Unfortunately, due to the limited access to FEL facilities, a thorough investigation depending on the base temperature and/or pump fluence was not possible. However, we were able to collect a partial dataset at another base temperature (117 K). Although the analysis of this dataset is limited and thus was not included in our original submission, because it has only a few delay points and each delay point was measured only once. However, we believe that it can still be used to provide additional insights for the generality of our results. Therefore, we have included the 'Results from the base temperature of 117 K' section and new supplementary figures in the SI as shown below.

"Results from the base temperature of 117 K

Experimental data with a base temperature of 117 K was collected and analyzed using the same protocol described in the "Methods" section of the main text. The results are summarized in Figures S7 and S8. Although the number of delay point and statistics of the data are limited, the results appear to be consistent with those of the measurement with a base temperature of 170 K. The difference intensities, both in the SAXS and WAXS regions, show similar shape and time-dependent trends to those of the measurement with a base temperature of 170 K, indicating a similar melting and recrystallization process occurring in the sample. The relatively smaller molten fraction and SAXS intensity at this temperature compared with the case of 170 K are consistent with the effect of the lower base temperature. Additionally, it is shown that the decay of the SAXS intensity is slower than that of the melt thickness, indicating coalescence between the adjacent domains.

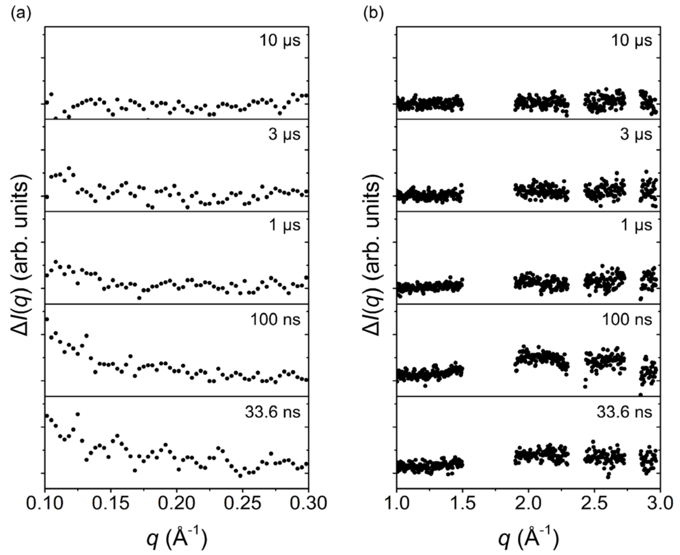

Fig. S7. The difference scattering intensities (black circles) in the (a) SAXS and (b) WAXS regions measured with the base temperature of 117 K.

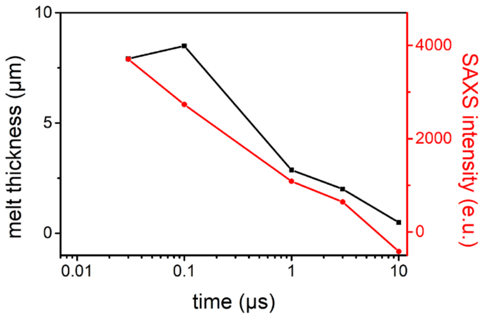

Fig. S8. The time-dependent change of the thickness of the liquid domain (black square) and the integrated intensity of the SAXS region of the difference scattering pattern (red circle) measured with the base temperature of 117 K."

In addition, we would like to note that the generality of our finding is supported by the comparison with the previous time-resolved IR study (at a base temperature of 270 K), as we discussed in the original manuscript. Specifically, the results from the previous IR study and our study are generally consistent, and differences such as the molten fraction can be well explained by the differences in the base temperature (170 K vs. 270 K). To further clarify those points, we have revised the following sentence in the manuscript:

From

"Once more, we consider this discrepancy to be caused by our lower base temperature (170 K vs. 270 K)."

To

"Once more, we consider this discrepancy to be caused by our lower base temperature (170 K vs. 270 K). This is also consistent with the results from our measurement at the base temperature of 117 K (see Supplementary Information for details). For example, only about 8 % of the molten fraction was found

at this condition.”

As the reviewer pointed out, in future studies, it is possible to tune the intensity of the pump pulse and it would indeed be a good way of investigating the nature of superheating phenomena more in detail. We have revised the following sentence in our manuscript to include this outlook.

From

“Finally, we note that by utilizing the experimental and analysis protocol established here, future experiments focused on the femtosecond time regime can reveal important structural details in the ice phase of the initial melting process, such as the mechanism of the formation of superheated water or the energy dissipation through the ice lattice.”

To

“Finally, we note that by utilizing the experimental and analysis protocol established here, future experiments focused on the femtosecond time regime and/or using the various pump fluences can reveal important structural details in the ice phase of the initial melting process, such as the mechanism of the formation of superheated water or the energy dissipation through the ice lattice.”

Further points include.

1) The error on the estimate od the temperature is probably of the order of the spreading of the points in Fig. 2b. This should be made clear by adding an error-bar. Even better, if would be nice to see a set of experimental data (as in Fig. 1d) but with the best "fit" liquid s(q) and with the liquid s(q) at, let's say, 10 kelvin higher and lower.

→ We have added error bars in the revised Fig. 2(b) as shown below. Note that the plot is now compared with the curve from the simulated heat dissipation. As the reviewer pointed out, the error on the estimated temperature is of the similar order as the spread of the data. To address this point more explicitly, we have revised the relevant sentence in the main manuscript as follows.

From

“The temperature rises from the base temperature (170 K) to ~350 K within our experimental time resolution and then decreases to the melting temperature (~270 K) within 10 μs.”

To

“While the estimated temperatures have relatively large errors, we can still observe the time-dependent trend. The temperature rises from the base temperature (170 K) to ~350 K within our experimental time resolution and then decreases to the melting temperature (~270 K) within 10 μs.”

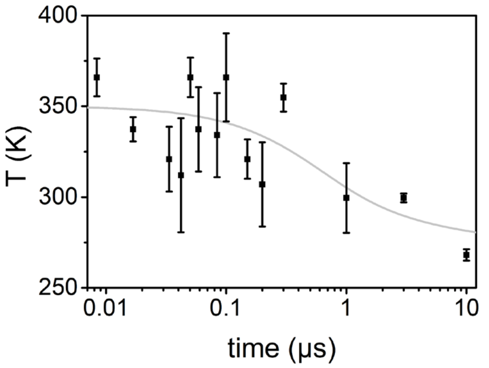

Fig. 2. (b) The estimated temperatures of liquid water at each time delay (black square) and the simulated temperature change by the heat dissipation (gray line).

As suggested by the reviewer, we have compared the experimental data with the fits at around ±10 K. Although the differences are very small as inferred from the large error bars of the estimated temperature, the sum of squared difference shows a clear minimum at around the best fit temperature.

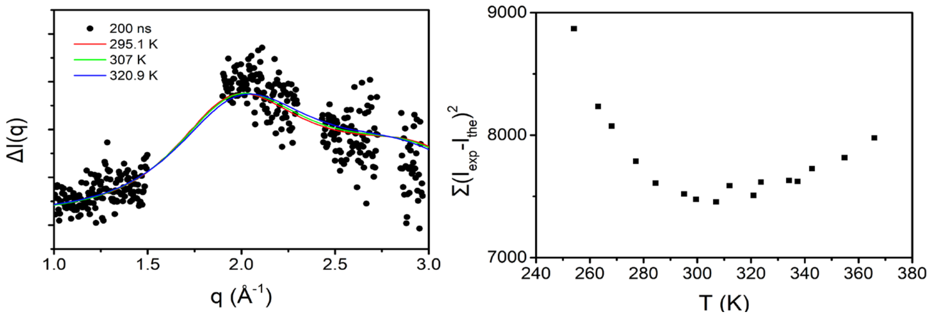

2) The fit of the SAXS data is not really perfect at very low q (Fig. 1c). The authors should comment on this imperfection. Does it arise from experimental error in the subtraction procedure ? Or does it signal a real problem with the functional form ?

→ As the reviewer pointed out, the fits of the SAXS data in some delays are not perfect. This is mainly due to the use of only one fitting parameter (radius of the liquid domain, *r*) that was adjusted freely during the fitting analysis. Other factors in equation 1 are fixed to values estimated from other analysis and the errors from those estimations are inherited to the fit of the SAXS data. We can in principle use *c* (scaling factor), N_p (number of the molten domain), and the shape of the molten domain (this will change the functional form as the reviewer mentioned) as fitting parameters, and the fit becomes much better if we adjust any one of these parameters freely together with *r*. However, using multiple free parameters will increase the risk of overfitting significantly. Thus, we chose to minimize this risk by minimizing the number of free fitting parameters. To explain this point more explicitly, we have revised a sentence in the main text as follows.

From

"We note that the only free parameter that was adjusted during the fitting analysis is the radius of the liquid domain, *r*."

To

"To minimize the risk of overfitting, we used only one free parameter (radius of the liquid domain, *r*) which was adjusted during the fitting analysis, while the other parameters are fixed to values estimated from the WAXS analysis."

3) Eq.1 is nothing more than the form factor of a sphere. Why not to say it directly in the text ? This would make it easier for the reader who is not an expert in scattering.

→ We agree with the reviewer. To explicitly mention this fact, we have revised the relevant sentence in the main text,

from

“If we assume molten domains as homogeneously distributed spherical particles in the ice medium, the experimental SAXS patterns at each time delay can be fitted with the theoretical SAXS pattern calculated by the following equation (See Method section for details):”

to

"If we assume molten domains are homogeneously distributed spherical particles in the ice medium, we can fit the experimental SAXS patterns at each time delay with the theoretical SAXS pattern calculated by using the form factor of a sphere (see Method section for more details):"

In summary, this is an excellent experimental study which does deserve publication in Nature Communication. The authors show that nowadays, with cutting edge experiments, it becomes possible to access the very short timescale of melting and crystallisation. Still, the authors should make a significant effort to better explain to the reader the generality of their findings and how general are the "numbers" they have been able to evaluate for the first time.

→ By following the reviewer’s valuable comments, we have revised our manuscript as described above. We believe that the manuscript now better addresses the generality of our new findings.

Reviewer #2:

This paper uses a combination of time-resolved WAXS and SAXS to reveal structural changes in the time range from 0.01 to 10 microseconds in the melting and re-crystallization processes of ice. The authors monitor the growth or disappearance of the liquid domain in partially melted ice. The obtained data by using the time-resolved X-ray scattering techniques are significantly valuable in understanding the unique nature of water. I think that the paper should be published. However, some modifications seem to be needed. My comments and questions are as follows:

→ We appreciate the reviewer's valuable comments and critical questions about our work.

1. WAXS data is expected to contain hydrogen bonding information. The authors extracted only the thickness of the liquid phase from the WAXS information. Is it possible to extract hydrogen bonding information?

→ As the reviewer pointed out, WAXS data in principle contain information about hydrogen bonding but extracting the pair-distribution function from the experimental data, here is not straight forward. More specifically, the data need to be Fourier transformed into the real space to get the radial distribution functions and it requires the data collection up to a very large q which is not possible at the current FEL with a limit in the accessible photon energy range. Therefore, our experimental data does not cover up to a q range sufficient for the Fourier transformation. Nevertheless, we agree that obtaining direct structural information on the changes in hydrogen bonding network during the melting process by the Fourier transformation of WAXS data would be a very good subject for a potential follow-up study, because the experimental protocol established here can be used at a beamline that can provide x-ray with very high energy. Such an experiment is becoming possible soon when the FELs can provide higher photon energies.

2. In Figure 2b, the dashed line could be misinterpreted as a theoretical value.

Is it possible to show it with S5?

→ This is a good point and we think that putting the simulated cooling curve instead is a great idea. By following the reviewer's suggestion, we have removed the guide for the eye from Fig. 2(b) and instead incorporated the simulated heat dissipation that is shown in Fig. S5 (see below). We observe that the time-dependent change of the temperature and the simulated cooling curve are in qualitatively good agreement. We have revised the figure caption and the relevant sentence in the main text as follows.

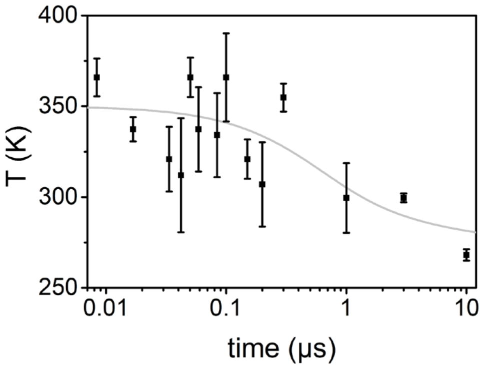

Fig. 2. (b) The estimated temperatures of liquid water at each time delay (black square) and the simulated temperature change by the heat dissipation (gray line).

From

"The timescale of the heat dissipation is simulated to be about 1 μs by using the thermal diffusivity of ice[31] and is qualitatively in good agreement with the value estimated from Fig 2(b)."

To

"The timescale of the heat dissipation is simulated to be about 1 μs by using the thermal diffusivity of ice[31] and is qualitatively in good agreement with the value estimated from the experiment as shown in Fig. 2(b)."

3. Error bars are necessary in Figs. 2(b) and 3(b).

→ We appreciate the reviewer for pointing this out. We have added error bars in the revised Fig. 2(b) and Fig. 3(b) as shown below.

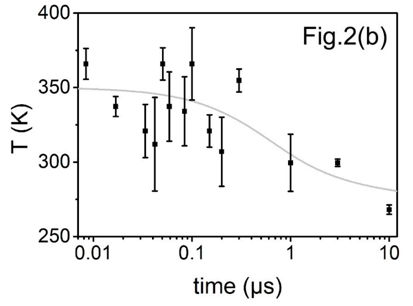

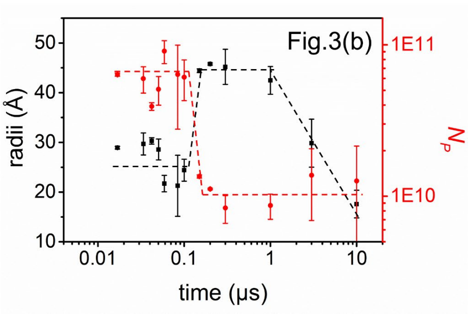

4. Fig.3 (a) shows the theoretical curves. Why are some spike-like noises seen?

→ The spike-like noises are indeed artifacts. Because we calculated the theoretical SAXS pattern numerically, finite step sizes for *r* and *q* were used and some small spikes came when *q* matches a specific period. To address the reviewer's concern and remove any potential misunderstanding from the readers, we have removed these artifacts by adjusting the step sizes and revised Fig. 3(a) accordingly as shown below. We note that this artifact in our previous calculation did not affect any of our results since the estimated sizes from the experimental data are all smaller than 10 nm (100 Å) and there were no spikes within this range.

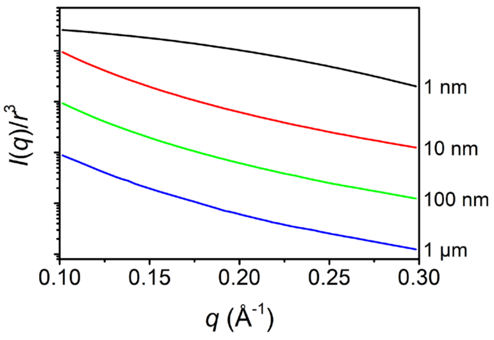

REVIEWERS' COMMENTS

Reviewer #1 (Remarks to the Author):

THe authors have properly taken into account all my previous requests. I am in favour of publishing the present version as it is.

Reviewer #2 (Remarks to the Author):

The revised paper has been improved in accordance with the reviewers' opinions. Therefore, I recommend that this paper is published.
